# Diversified Talent Cultivation Mechanism of Early Childhood Physical Education Under the Full-Practice Concept – Oriented by Preschooler Mental Health and Intelligent Teaching

**DOI:** 10.3389/fpsyg.2020.593063

**Published:** 2021-01-15

**Authors:** Nina Wang, Mohd Nazri Bin Abdul Rahman, Megat Ahmad Kamaluddin Bin Megat Daud

**Affiliations:** ^1^Faculty of Education, University of Malaya, Kuala Lumpur, Malaysia; ^2^Department of Educational Psychology and Counseling, Faculty of Education, University of Malaya, Kuala Lumpur, Malaysia; ^3^Department of Educational Management, Planning and Policy, Faculty of Education, University of Malaya, Kuala Lumpur, Malaysia

**Keywords:** early childhood physical education, talent cultivation mechanism, full-practice concept, artificial intelligence equipment, preschooler mental health

## Abstract

In order to improve early childhood physical education, in this study, the talent cultivation mechanism for undergraduates was explored under the “full-practice” concept, oriented by preschooler mental health. First, from the perspective of preschooler psychology, the mechanisms of ability training and talent cultivation for undergraduates majoring in early childhood education were explored under the “full-practice” concept. Considering that the physical, psychological, and intellectual development of preschoolers shall follow the rules of physical education, and current early childhood education mainly focuses on intelligence education in China, early childhood physical education was analyzed further in this study. By investigating the undergraduate majors of early childhood education in Henan University, this study first summarized the current problems in early childhood education systems in universities. Secondly, combined with the form of physical education in kindergartens, strategies for talent cultivation and curriculum setting of early childhood physical education majors in colleges and universities were proposed. Finally, from the perspective of innovation and diversification of training forms, the cultivation of early childhood educators’ physical education ability was analyzed at multiple levels and multiple objectives, and the integrated training system of early childhood education talents was constructed. The results show that, among all the courses for early childhood education major, compulsory courses account for 81.2% and optional courses account for 18.8%. In addition, a survey on undergraduates’ attitudes toward the curriculum of their major demonstrates that 81.2% of the undergraduates thought that the range and content of practical courses should be increased, indicating that undergraduates majoring in early childhood education are dissatisfied with the current curriculum system, and they have an increased demand for practical courses. Correspondingly, it is vital to build and improve on the early childhood physical education. In terms of its talent cultivation, the “full-practice” concept helps combine theory with practice to improve the effectiveness of education and teaching, pushing forward the reform of the education system. Meanwhile, data- and intelligence-oriented teaching will become the new direction of modern sports development, as well as an important link for tracking and monitoring children’s sports teaching in China. Through the continuous introduction of wearable artificial intelligence (AI) products, real-time monitoring of children’s physical conditions can be realized, which helps improve the effectiveness of early childhood physical education.

## Introduction

Physical education stimulates enthusiasm and creativity in people. Its content is closely correlated to energetic behaviors in the age characteristics of preschoolers (Mozolev et al., 2019). However, physical education is not only the most easily neglected link but also the most important link in early childhood education. Early childhood education combines the care and education of preschoolers. While carrying out a comprehensive and harmonious education for preschoolers, physical education must be prioritized to make preschoolers healthy and promote their all-round development. In order to emphasize the primary position of physical education in preschoolers, it is necessary to oppose the emphasis on intellectual education and neglect of physical education in early childhood education (Lander et al., 2017). Considering the physical development of preschoolers and the requirements of the current age, teachers may guide preschoolers to cooperate through physical activities, which can facilitate the physical and mental development of preschoolers and stimulate the interests of preschoolers in learning (Cheung, 2020).

Early childhood is a critical stage; education at this stage will be the foundation for education at later stages. As a critical field of early childhood education, physical education is significant for both physical fitness and as a foundation of preschoolers’ lifelong physical education in schools (Bruijns et al., 2019). According to the *Guidelines for Kindergarten Education*, kindergarten education is an essential component of elementary education, so protecting the lives and promoting the health of preschoolers are the priorities. Kindergarten teachers should guide children to participate in a variety of games and activities, thereby helping them experience and enjoy companionship (Ourdaa et al., 2017). Physical education for preschoolers also helps complete the education system, establish novel concepts for early childhood education, and promote the rapid development of education science. In addition, appropriate educational methods are of significance for stimulating preschoolers’ interest in sports and for their all-round development (Petrenko, 2016). It is thus important for teachers to develop cooperative behaviors through early childhood physical education. Therefore, for early childhood education, improving the physical education ability of teachers is the current focus of talent cultivation. Nevertheless, the demand in modern physical education for teaching auxiliary equipment is continuously increasing. In the traditional children’s physical education teaching model, the application of wearable AI products improves the efficiency of the teaching process of teachers and students, and greatly saves on communication energy and time between teachers and students through more convenient and faster electronic information transfer, so to some extent it plays a role in improving the quality of physical education.

To summarize, it is critical to develop the early childhood physical education for elementary education. Meanwhile, the cultivation of teachers determines the advancement of education reform and the implementation of the strategy of “reinvigorating the country through science and education.” To realize diversified talent cultivation in the early childhood physical education field, this study explores the mechanisms of ability training and talent cultivation for undergraduates majoring in early childhood education from the perspective of preschooler psychology under the full-practice concept. Hopefully, early childhood physical education can be improved. The discussion and analysis not only optimizes the theoretical system of preschool education majors in colleges and universities, but also explores the design of the characteristic early childhood physical education curriculum, which has positive significance for promoting the diversified and comprehensive development of children, and provides reference for the cultivation of undergraduate’s practical talents in preschool education.

## Literature Review

In the 1,980s and 1,990s, flexible and rich teaching methods were used to foster children’s trust in others and to help them learn to express their emotions appropriately. Sexual education was also added; mental health education was integrated into classroom games, group activities, and other courses. Teachers were required to note the mental health of children. Gradually, the form of education for mental health became more flexible and diverse, and the value of games was emphasized. Parent-child classrooms and team activities were utilized in mental health education. Such activities were implemented by focusing on integrity and comprehensiveness. At that time, there was a wealth of research on the individualization of kindergarten mental health education, and research on game theory had made some progress. Pasek et al. (2020) discussed the importance of mental health education and pointed out that the three directions for the development of mental health education include orientation, intervention, and integration. Emphasis was placed on the diagnosis, evaluation, and psychological counseling of children, and integration of special needs children into regular kindergartens, with an emphasis on providing developmental opportunities for each child.

With new comprehensions and requirements for the education of preschoolers from a psychological perspective, there is also a more specific and comprehensive direction for the cultivation of talent for early childhood education professionals and students. In the exploration of the teaching process, Chinese scholar Qin proposed the teaching concept of “full-practice,” that is, all aspects of the practice elements should be extended throughout time, the space should be comprehensively expanded, and the content should be fully integrated. This concept should be holistically infiltrated and fully integrated into the curriculum system (Qin, 2006). Among them, constructivist learning theory, situational learning theory, and the neural construction theory of cognitive neuroscience are the most important sources of thought. The staged practice teaching in teacher education includes multi-objective training, several stages in time, space, and type practice teaching bases at different levels, and staged evaluation. Frank et al. (2018) proposed that the curriculum mode under the full-practice concept included the implementation of practice courses, the improvement of the quality of practice courses, the participation of all staff in practice, and the strengthening of off-campus cooperation.

Different early childhood education institutions have varied educational purposes and teaching contents. Teaching activities are generally divided into eight aspects: cognitive, operational, mathematical, physiological, musical, natural, social, and game. Early childhood education aims to cultivate children’s creative ability. Kindergartens advocate that “teachers should not systematically impart knowledge to children in advance.” Kindergartens are not schools. Reading, writing, and calculation should be taught in primary schools, while fostering children’s creativity is what early childhood education should be about. However, the current educational curriculum lacks contemporary features in the training of early childhood sports personnel. The curriculum design pays too much attention to the teaching of professional knowledge and ignores the cultivation of extensive knowledge and the hidden curriculum, which greatly affects the cultivation of early childhood education professionals.

In summary, research on the training mechanism for early childhood education professionals mainly focuses on the training of preschool kindergarten teachers. However, there is a lack of research on the training mechanism to meet the diversified talent needs and regional development. Most of the previous achievements are part of the exploration of the educational operation mechanism. The corresponding policy guarantees are less involved, and there is a lack of a system suitable for the training of diverse talents for early childhood education. Therefore, there is an urgent need to improve the quality of early childhood education personnel training, as well as improving the curriculum and guaranteeing the diversity of preschool teachers in universities. Guided by preschooler psychology, new ideas are put forward for the educational model of students majoring in early childhood education. From this, the quality of talent training for undergraduates majoring in early childhood education will be ultimately improved by integrating the concept of full-practice education.

## Materials and Methods

### Preschooler Mental Health-Based Early Childhood Education

**FIGURE 1:**
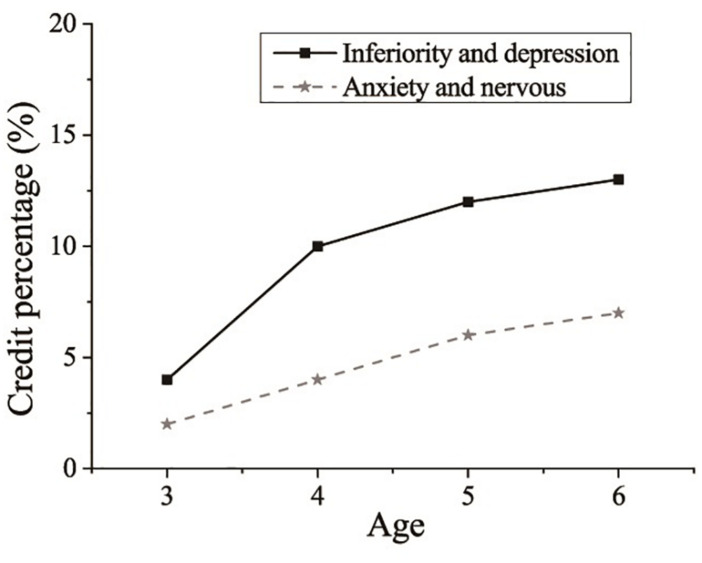
Results of a mental health survey among preschoolers aged 3–6 years old.

Mental health refers to a state of mental function, which represents the coordination and unification of internal psychology and the external environment. For preschoolers, their psychological characteristics are different from those of adults. However, no unified conclusions on the mental health of preschoolers have yet been reached. Statistics show that in 22 cities in China, the detection rate of preschoolers’ behaviors reached 12.97%. According to a mental health survey among 3,000 preschoolers aged 4–5 years old done by the Shanghai Mental Health Center, 8.8% of the participants have unhealthy habits, 11% suffer from inferiority and depression, 5.8% suffer from anxiety and tension, and 22% have a strange temperament (Smal, 2016). The forms of sports activities in kindergartens are shown in Table 1. The results of a mental health survey among preschoolers aged 3–6 years old in Shanghai are shown in Figure 1. This shows that the mental health education of preschoolers in China cannot be ignored.

**TABLE 1:** Forms of physical education activities in kindergartens (*n* = 22).

	Morning exercises	Physical education courses	Physical exercises between classes	Outdoor sports	Sports meeting	Recreational activities
Number of kindergartens	18	10	20	22	13	15
Proportion (%)	81.8%	45.5%	90.9%	100%	59.1%	68.2%

According to the United States Zero To Three Task Force, preschooler mental health refers to the emotional-social-behavioral health of preschoolers (respectively, in the family, community, and the living environment of preschoolers); specifically, it refers to the ability of preschoolers to experience, regulate, and express their emotions between the ages of 0–6. What needs clarifying is that the standards for preschooler mental health will change with social development and cultural progress. Therefore, preschooler mental health should be based on actual situations (Houser et al., 2019; Zimmo et al., 2020).

In fact, children still have pressure and anxiety, and younger children tend to have more mental health problems. These pressures and anxieties come from improper family education, heavy “tasks” imposed by kindergartens, or are influenced by social interaction. In the long run, children’s psychological pressure and anxiety cannot be released, which is not conducive to the healthy growth of children. Therefore, it is necessary to set up mental health education activities in kindergartens. Good psychological education can promote the healthy growth of children and play an important role in children’s early childhood; the moral construction of preschoolers can promote the education of children, so that children can grow up in a healthy, safe, and harmonious environment. In a broad sense, preschooler mental health includes the joint intervention of schools, families, and communities. For the intervention of schools, teachers divert the negative emotions of preschoolers through psychological theories and strategies according to the characteristics of preschoolers’ mental and physical development. After the mental problems of preschoolers are addressed, their harmonious and comprehensive development can be promoted, and the education quality can be improved in a comprehensive manner (Dallolio et al., 2016). In a narrow sense, preschooler mental health refers to mental health education activities in kindergartens.

The specific content of preschooler mental health education needs to reflect the goals of education. The content setting must not only be based on the understanding level and development needs of preschoolers but also consider the requirements of social change and development on preschoolers. According to current research progress, the contents and the goals of preschooler mental health education are summarized as follows: first, to help children learn simple self-adjustment methods of emotions; second, to enable children to master the ability to conduct safe social interactions; and third, in the process of mental health education, to help young children develop good behavioral habits. To achieve the above goals, corresponding requirements are raised for educators engaged in early childhood education (McKenzie et al., 2016; Ng et al., 2020). First, early childhood educators should hold a modern concept of early childhood education and actively create a relaxed and harmonious educational environment. Second, educators should integrate field-specific mental health education in knowledge teaching. Finally, educators must emphasize personalized education, and mental health education should value the personality cultivation and development of preschoolers. In addition to daily education, special mental health courses can also be set up to provide targeted analysis and education of mental problems. Cooperation between family education and school education must also not be ignored. Educational resources from multiple parties can be combined to exert a synergistic effect of mental health education.

### Application of “Full-Practice” Concept in Talent Cultivation Among Undergraduates

Early childhood education must not only focus on the behavioral education of preschoolers but also value the nature of preschoolers at this age (Haegele et al., 2017; Weaver et al., 2018; Lehto et al., 2019). As a result, early childhood physical education should also be valued. For preschoolers, physical activity is a planned and organized educational activity, which mainly promotes the physical and mental development of children through the guidance and training of their physical movements, while enhancing their physical fitness. In addition, physical education helps preschoolers develop their intelligence, which is conducive to the coordinated development of body and mind (Chaput et al., 2017). The teaching philosophy of a university proposes that the talent cultivation for undergraduates majoring in early childhood education should meet the needs of social development and modern education reform, thereby matching the talent cultivation with the needs of the social market. Therefore, the ultimate objective of a university is to develop application-oriented talents.

**FIGURE 2:**
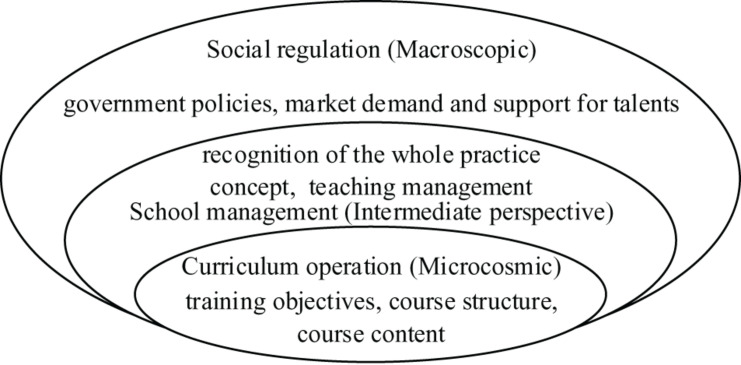
Talent cultivation mechanism for undergraduates majoring in early childhood education in China.

Currently, the theoretical knowledge of teaching is often disconnected from practical teaching. Some developed countries have begun to change the traditional education model for teachers, build a practice-oriented teacher education curriculum system, and perform useful practical attempts. The Japan Education University Association first proposed the concept of “educational practical experience” to highlight the practical orientation of teacher education courses and enhance the practical ability of teachers (Figueroa et al., 2019). In China, the talent cultivation of early childhood education is mainly controlled and managed from three aspects: social regulation, school management, and teaching operation. The structure of them is shown in Figure 2. Schools should take the training goal as the orientation, carry on the overall planning, set up the training focus and teaching goal, implement the goal of each stage into the curriculum of each stage, and establish the gradual curriculum mode. In addition, the curriculum of early childhood education at an undergraduate level should be guided by *The Professional Standards for Early Childhood Teachers*, and the quality of teachers should be developed in various courses. Colleges and universities provide convenience for further study of academic qualifications and skills’ improvement training for early childhood education institutions, and guide the development of scientific research projects, teaching reform projects, or special work; early childhood education institutions provide colleges and universities with practical opportunities such as practical teaching, investigation and research, social practice, professional internship, and teaching and scientific research experiment, and the two sides establish a deeply integrated cooperative relationship.

The “full-practice” concept and its teaching methods are scientific and feasible forms of teacher education. It can infiltrate practical teaching throughout the learning process of undergraduates, which is consistent with the current development trend of practical talent cultivation (Togo and Caz, 2019). Recently, with the increasing development of education in China, national policies have also greatly supported the talent cultivation of undergraduates majoring in early childhood education. From national and social perspectives, the reform of education talent cultivation is promoted by formulating courses and teaching materials for early childhood education majors. In the *Notice on Increasing Financial Investment to Support the Development of Early Childhood Education* issued by the Ministry of Finance and the Ministry of Education in 2011, the training plan for preschool teachers have been scientifically formulated, the training model has been innovated, the training system has been improved, and the general quality and professionalism of preschool teacher education have been comprehensively improved (Jayachitra, 2018).

From the perspective of school regulation, theoretically, during the cultivation process of early childhood education professionals, the simple explanation in traditional teaching methods should be transformed. After teaching theoretical knowledge, students should be allowed to diversify their thinking, practice their knowledge through operation, and thereby progress through reflection (Lehto et al., 2018). In colleges and universities, the biggest problem in the current teaching model is that there is no innovation in the management system. Most of them are the same, and there is no corresponding development plan for students of different majors. For undergraduates of early childhood education majors, the long-term development of this major needs to be based on the educational regulations of colleges and universities. For the old and rigid rules and regulations that do not conform to the development of the times, corresponding reforms are needed. For example, the curriculum is not based on the specific needs of students; also, it lacks corresponding practical teaching management, guidance in career development planning, and targeted incentives for students. Therefore, students have low professional recognition (Yan, 2019). In addition, the faculty strength in the guarantee mechanism of colleges and universities is the prerequisite for the smooth development of talents. With the continuous increase in the faculty strength of early childhood education majors, the ratio of teachers with Master and Doctorate degrees is increasing. However, deficiencies also exist in learning from the practice experience of high-quality kindergarten teachers. Consequently, undergraduates cannot fully understand the practical problems in early childhood education. In addition, according to the needs of market development, an early childhood education major has gradually penetrated early education institutions and other fields. Therefore, it is essential for universities to develop comprehensive and diversified training plans for undergraduates, and it is necessary to strengthen the composite faculty strength of colleges and universities (Tosten et al., 2017; Matwiejczyk et al., 2018).

Finally, from the micro aspects of curricular setting and operation, the current curriculum is mainly composed of four modules, i.e., professional courses (compulsory courses), optional courses, general courses, and practical courses. The compulsory courses account for the majority of the curriculum structure, which mainly involves the teaching of theoretical knowledge. Meanwhile, the arrangement of practical courses is at a disadvantage, and the teaching form is superficial. The practical courses of many colleges and universities are not strong enough in inspections and examinations, causing undergraduates to undervalue the practical courses (Hu et al., 2017; González-Valero et al., 2019; Malchrowicz-Mośko et al., 2019).

### Application of Wearable AI Products in Children’s Physical Education

The hardware core of wearable artificial intelligence products is sensor and wearable technology, which is used to transform physiological information. The core of software is wireless network transmission technology and data statistics processing technology. Wearable AI products can collect the whole process of human physiological parameters in the process of exercise and provide targeted individual exercise prescriptions and nutrition suggestions for exercisers through big data analysis systems.

The development of this field in China is still in its infancy due to the restrictions on the research and development (R and D) of core hardware and software. In recent years, the R and D of related products has also made great progress. The query and statistics of network resources suggest that the patent application of wearable AI products has been greatly increased in recent years. Wearable AI sports products are a kind of portable health equipment that can record the physiological parameters of a human continuously for a long time. According to the functions, wearable AI sports products can be classified into sports health, health management, and somatosensory control. The main technical features of wearable AI sports products includes speech recognition, sensors, low-power interconnection technology, high-speed internet, and cloud computing, which can non-invasively monitor the health of the wearer daily. It is characterized by intelligent display results, convenient operation, long-term continuous use, and wireless data transmission. Wearable AI products are the trend and future of the information technology revolution. Its pertinence, real-time, and security monitoring characteristics are of great significance for health services and sports training.

### Experimental Contents and Research Methods

To explore the current talent cultivation mechanism of undergraduates majoring in early childhood education, this study performs a survey and investigation in Henan University that has an undergraduate major for early childhood education. Through questionnaires for 2015-entered undergraduates majoring in early childhood education, the professional curriculum and teaching operation are used as a reference basis for the undergraduate cultivation of early childhood education professionals. The early childhood education major is listed as one of the two key majors in the priority development and characteristic construction of University A. In recent years, it has increased its investment in construction of human resources, financial resources, and other resources. It developed rapidly and is a representative of emerging universities that train early childhood education professionals. It mainly includes the number of theoretical required courses and practical courses, the intention of students to pursue theoretical and practical courses, and the demand of students for practical courses. The reliability and validity of the questionnaire is tested to ensure the measurement ability and the validity of the results.

In this study, students in two 2015 classes were selected for investigation. A total of 110 questionnaires were distributed, and 106 questionnaires were retrieved. Also, five questionnaires with incomplete information were excluded. Therefore, a total of 101 valid questionnaires were collected. The effective rate of questionnaires was 91.8%. Among the 101 survey subjects, there were 26 boys and 75 girls, aged 20 to 21 years.

The content of the questionnaire involves the curriculum of early childhood education and the practical teaching and employment guidance. The content of the questionnaire was evaluated previously to ensure its validity. To have a preliminary understanding of the current development of physical education in kindergartens in China, this study selected 22 kindergarten teachers in three districts of Henan Province, China as the survey objects by the random sampling method, in which nine were public kindergartens and 13 were private kindergartens. A total of 76 questionnaires were issued and 76 were recovered, of which 72 were valid questionnaires, and the valid rate of the questionnaires was 94.7%. In this study, statistical software SPSS 26.0 and EXCEL were used for statistical analysis of the data. In addition, interviews were conducted on some issues according to the particularities of the survey subjects.

## Results

### Survey Results of Current Early Childhood Education of Undergraduate Education

**FIGURE 3:**
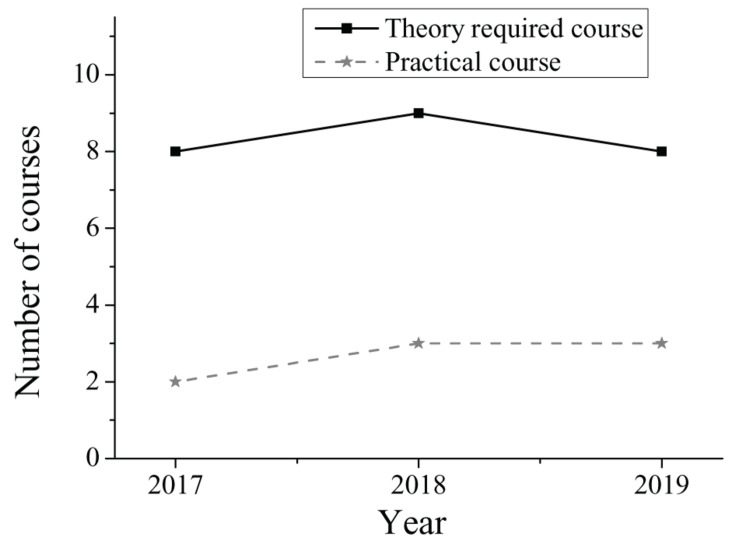
Compulsory courses and practical courses of junior students in 2017–2019.

**FIGURE 4:**
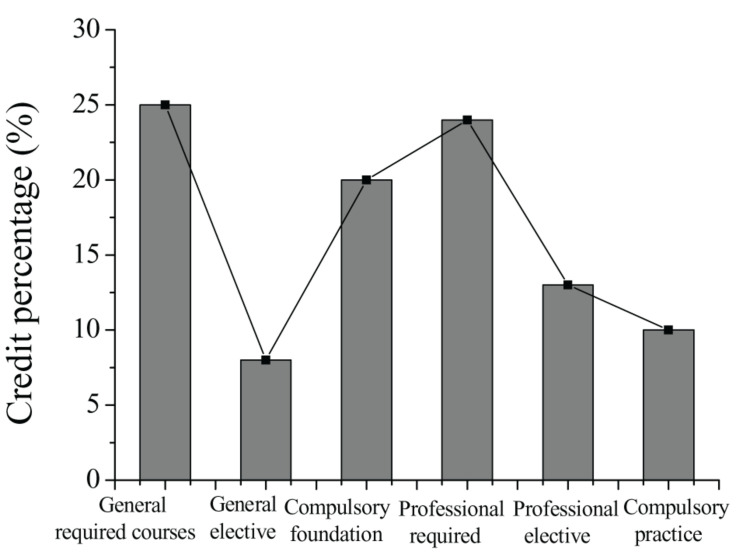
Curriculum of courses in University A.

The teaching philosophy of University A proposes that the talent cultivation for undergraduates majoring in early childhood education should meet the needs of social development and modern education reform, thereby matching the talent cultivation with the needs of the social market. Therefore, the ultimate objective of University A is to train application-oriented talents. The changes in compulsory courses and practical courses for junior students in 2017–2019 are shown in Figure 3. By investigating the curriculum of early childhood education majors, it was found that compulsory courses account for 81.2% and optional courses account for 18.8%. This shows that junior students of University A have less autonomy in selecting courses; there is not much room for them to reflect their intentions. The curriculum of courses is shown in Figure 4.

**FIGURE 5:**
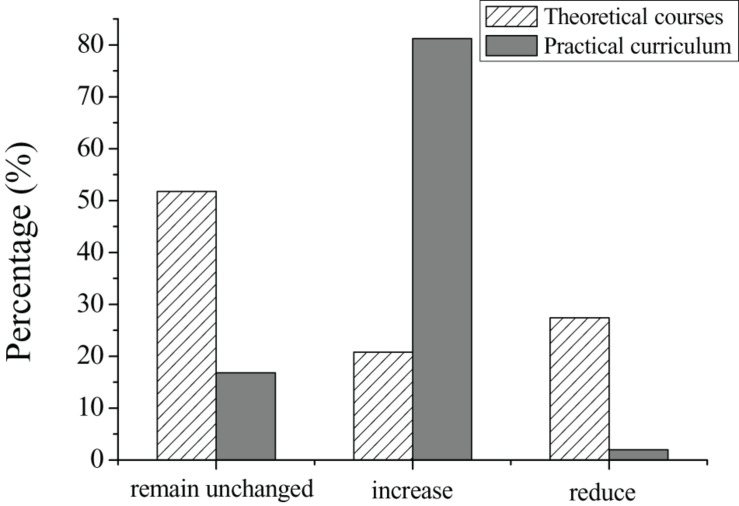
Intentions of undergraduates on the curriculum of theoretical and practical courses.

As shown in Figure 4, the credits for the practical courses are 10%. Through investigation, it was found that the practical courses are mainly arranged during the freshman year through to the junior year of the students; practical education is performed by interning in kindergartens or early education institutions. A total of 85 (84.1%) questionnaires show that the time arranged for practical courses of junior students in school is only 4–5 weeks, which shows that the practice time for undergraduates majoring in early childhood education is insufficient. For this situation, the intentions of students in curriculum settings are also investigated. The results show that for current theoretical courses, 51.8% of students believe that the curriculum should maintain the *status quo*, 20.8% of students believe that the curriculum items and contents should be increased, and the remaining 27.4% of students believe that the theoretical courses should be appropriately reduced. From the perspective of students’ attitude toward practical courses, 16.8% of students believe that the practical courses should remain unchanged, while 81.2% of students believe that the categories and contents of the practical courses should be increased, and only 2.0% of students believe that the curriculum of the practical courses should be reduced. The intentions of undergraduates on the curriculum of theoretical and practical courses are shown in Figure 5. As shown in Figure 5, undergraduates majoring in early childhood education are dissatisfied with the current curriculum system, especially the increase in demand for practical courses.

**FIGURE 6:**
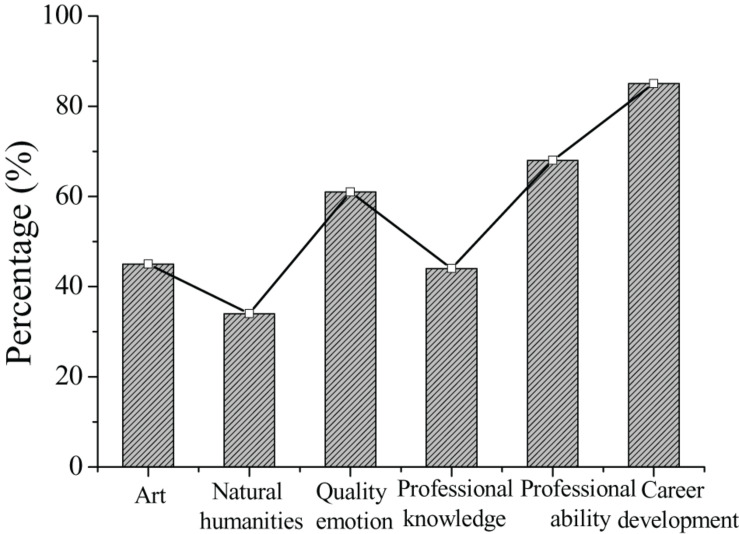
Intentions of students on adding experimental courses.

As shown in Figure 6, a total of 82 (81.2%) students who participated in the survey thought that the arrangement of practical courses should be increased. Furthermore, the types of experimental courses that students wish to increase are investigated through the survey. The results of the survey are shown in Figure 4. The results show that students have the highest demand for practical courses in career development planning and teacher ability development, accounting for 85.4 and 70.0%, respectively. These results show that students majoring in preschool education pay more attention to practical teaching than theoretical teaching. Students believe that through practical teaching, they can experience first-hand the various problems faced by children in the process of growth, which is helpful for students of this major to solve unexpected problems in their work.

### Analysis of the Physical Education in Kindergartens

According to the questionnaire survey, in Henan Province, physical exercises in kindergartens mainly include morning exercises, physical exercises between classes, outdoor activities, and art performances. The form of art performances is mainly dancing. The survey has found that all schools have set outdoor sports activities, followed by physical education in the form of physical exercises between classes and morning exercises, accounting for 90.9 and 81.8%, respectively. Only 10 kindergartens (45.5%) specialized in physical education courses. According to the feedback from kindergarten teachers, the major reasons that it is not common to offer physical education courses in kindergartens are: (1) teachers lack professional knowledge and cannot complete the tasks of physical education in a standard manner to realize the purpose of physical education; and (2) the physical fitness and tolerance of preschoolers are limited, and physical education is questioned for safety concerns.

The time for outdoor activities of preschoolers should be no less than 2 h a day, in which physical activities should be no less than 1 h. This survey has found that kindergartens in Henan Province have met the time standards for physical activities. In addition, 16 of the 22 kindergartens have ensured that the average daily physical activity time reaches 1–2 h, accounting for 72.7%. There are five kindergartens with less than 1 h of daily physical activity. Kindergartens with an average of less than 1 h of daily physical activity are restricted by the area of the campus or the excessive school schedules; insufficient attention is paid to physical activities. Sufficient daily physical activity time is a critical factor to ensure the physical development of preschoolers. The combination of activities and physical education can improve the coordination ability and team spirit of preschoolers.

### Talent Cultivation Strategies for Undergraduates Majoring in Early Childhood Physical Education

**FIGURE 7:**
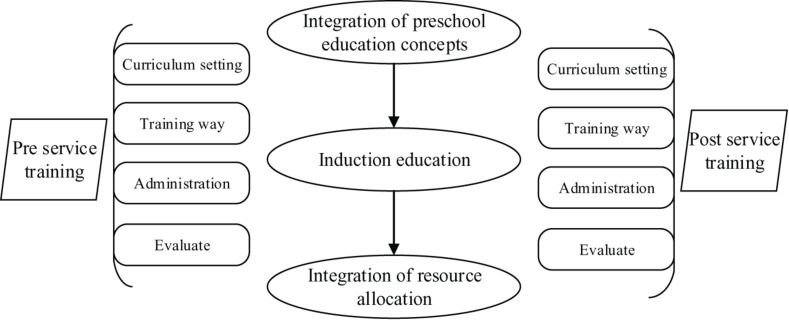
Integrated development of physical educating ability for undergraduates majoring in early childhood physical education.

In China, the degrees of education for preschool teachers include Master’s degree (graduate), Bachelor’s degree (undergraduate), and Associate degree (college student; Poppe et al., 2018; Barbosa Filho et al., 2019). Although they have the same task of cultivating preschool teachers, each education level has a different emphasis on training talents due to different academic systems, student sources, and training goals. For undergraduates, they should actively apply and practice the theories. Universities and colleges should cultivate diverse teaching staff who are familiar with early childhood education, teaching, and management. Under the influence of diversified theories, teachers should correspondingly stimulate their awareness of cultivating physical education capabilities, especially to promote the combination of theory and practice through practical links. The talent cultivation strategies for undergraduates majoring in early childhood physical education are mainly considered from the following aspects:

(1) Professional Ability Training in Physical Education Practice: The application of situational teaching should be emphasized in the physical education practice process, which not only enables students to participate in teaching as much as possible but also cultivates the practical ability of students. The main body of practical teaching is students, while teachers are mainly responsible for guidance. After the teachers have completed the standardized demonstration, students should practice and summarize the problems to improve their professional ability. Also, students are encouraged to form interest groups and organize skill competitions of physical education, thereby allowing students to develop better through the competitions.
(2) Construction of an Integrated Training System for Early Childhood Education Talents: The training of preschool educators’ physical education ability is a multi-level, multi-objective, and systemic task, which is an integrated process. Integrated education and training help undergraduates perfectly link up their education and employment, forming a situation of complementary advantages. In addition, through integrated education, students can obtain diverse knowledge from society as soon as possible, thereby improving their comprehensive development ability. The integrated development of physical educating ability for undergraduates majoring in early childhood physical education is shown in Figure 7. The integrated talent training model is mainly divided into three aspects: the integration of early childhood education concepts, onboarding education, and resource allocation integration. It starts from the four links of course setting, training methods, training management, and effect evaluation to complete the systematic talent training process.

(3) Innovative and Diversified Training Forms: Whether from a macro or a micro perspective, it is required to integrate innovative ideas into the talent cultivation models, take teacher development as the goal, and attach importance to the problem of teaching model and quality control of education talents. Meanwhile, in the process of promoting talent cultivation, students should value their self-development planning and awareness based on skills and knowledge. Students choose courses according to their own needs, including not only courses for preschooler behavioral habits education but also courses for preschooler hobbies, such as music and art. For skill training, innovative concepts should be introduced into advanced physical education theories. It is generally believed that kindergarten-based training can promote the development of physical education activities. Both the single training form and the combination of multiple training forms can fully reflect the pertinence and effectiveness of kindergarten-based training.

## Discussion

The development of early childhood education and the growth of preschoolers in a good educational environment are the educational expectations of the entire society, and is also the direction of long-term education reform of China in the future (Mendoza-Núñez et al., 2017; Sun et al., 2019; Dang et al., 2020). Therefore, in order to realize diversified talent cultivation in the early childhood physical education field, this study explored the mechanisms of ability training and talent cultivation for undergraduates majoring in early childhood education from the perspective of preschooler psychology under the full-practice concept. Hopefully, early childhood physical education can be improved.

Through the investigation and study of undergraduates in University A majoring in early childhood education, the teaching status of undergraduates majoring in early childhood education were understood and the deficiencies in curriculum setting and students’ demand for course content were identified. The survey was utilized as a reference basis for the training of undergraduate talents majoring in early childhood education. The results show that of all the courses for the early childhood education major, compulsory courses account for 81.2% and optional courses account for 18.8%. This shows that students have less autonomy in selecting courses; there is not much room for them to reflect their intentions. Also, 81.2% of the undergraduates thought that the range and content of practical courses should be increased. This shows that undergraduates majoring in early childhood education are dissatisfied with the current curriculum system, especially with the increase in demand for practical courses. To address the current situation, the innovative-practical education and teaching strategies were proposed for the talent cultivation of early childhood physical education; also, requirements for talent cultivation were put forward at the macro and micro perspectives. In the process of physical education practice teaching, it is necessary to focus on the application of situational teaching, which can not only enable college students to participate in teaching as much as possible, but also cultivate their practical ability. By integrating the innovative concept into the talent training mode and taking teacher development as the goal, this study combined various training forms, and applied them to fully reflect the pertinence and effectiveness of kindergarten-based training.

## Conclusion

Early childhood physical education is vital in the early childhood education system and lifelong physical education system. Therefore, society should value early childhood physical education and the talent cultivation of physical education. The talent cultivation of early childhood physical education takes the “full-practice” concept as the basis, promotes the diversified talent cultivation of undergraduates majoring in early childhood education, and helps combine theory with practice to improve the effectiveness of education and teaching, pushing forward the reform of the education system. Wearable AI products have the basic characteristics of portability, visibility, security, and using science and technology. In the information age, the application function of wearable AI products in physical education is becoming more powerful, and has great development potential for modern children’s physical education. As the key place for talent cultivation, colleges and universities should adjust from the aspects of faculty strength and curriculum setting to optimize the talent cultivation system. Although a more detailed survey has been conducted, the reliability and validity of the questionnaire survey still needs to be improved due to the lack of personal abilities. In addition, in terms of related suggestions, the current talent training mechanism involves a wider scope, so the content of specific mechanisms still needs to be explored more deeply. Although a more detailed survey and analysis has been done in this study, there are still some deficiencies. For example, the reliability and validity of the questionnaire survey needs to be improved, and there are certain cognitive biases in the answers of the students surveyed, which will affect the results.

## Data Availability Statement

The raw data supporting the conclusions of this article will be made available by the authors, without undue reservation.

## Ethics Statement

The studies involving human participants were reviewed and approved by the University of Malaya Ethics Committee. The patients/participants provided their written informed consent to participate in this study. Written informed consent was obtained from the individual(s) for the publication of any potentially identifiable images or data included in this article.

## Author Contributions

All authors listed have made a substantial, direct, and intellectual contribution to the work, and approved it for publication.

## Conflict of Interest

The authors declare that the research was conducted in the absence of any commercial or financial relationships that could be construed as a potential conflict of interest.
